# Metabolic alterations in the right anterior insula among patients with cirrhosis without overt hepatic encephalopathy: a magnetic resonance spectroscopy study

**DOI:** 10.3389/fneur.2023.1291478

**Published:** 2024-01-11

**Authors:** Nao-Xin Huang, Hui-Wei Huang, Qiu-Yi Dong, Yu-Lin Wen, Dan Li, Jian-Qi Li, Hua-Jun Chen

**Affiliations:** ^1^Department of Radiology, Fujian Medical University Union Hospital, Fuzhou, China; ^2^Shanghai Key Laboratory of Magnetic Resonance, School of Physics and Electronic Science, East China Normal University, Shanghai, China; ^3^Department of Gastroenterology, Fujian Medical University Union Hospital, Fuzhou, China

**Keywords:** ammonia intoxication, cirrhosis, cognitive dysfunction, magnetic resonance spectroscopy, right anterior insula

## Abstract

**Purpose:**

We investigated metabolic alterations in the right anterior insula (rAI) in cirrhotic patients and determined its association with patients' cognitive dysfunction.

**Methods:**

In this study, 31 healthy controls (HCs) and 32 cirrhotic patients without overt hepatic encephalopathy participated. Both blood ammonia level and Child-Pugh score were measured. The psychometric hepatic encephalopathy score (PHES) was used to evaluate cognitive function. ^1^H-magnetic resonance spectroscopy (MRS) data located in the rAI were recorded on a commercially available 3T magnetic resonance imaging scanner. The ratios of metabolites were measured, including N-acetylaspartate (NAA)/total creatine (tCr), glutamate plus glutamine (Glx)/tCr, myo-inositol (mI)/tCr, and total choline (tCho)/tCr. We adopted the non-parametric Mann–Whitney *U*-test for intergroup comparison of metabolic ratios. To determine the association between metabolite concentration and clinical parameters, we performed Spearman correlation analyses.

**Results:**

Patients with cirrhosis performed worse on PHES in comparison with HCs (*P* < 0.001). Patients with cirrhosis had significantly decreased mI/tCr (0.87 ± 0.07 vs. 0.74 ± 0.19, *P* = 0.025) and increased Glx/tCr (1.79 ± 0.17 vs. 2.07 ± 0.29, *P* < 0.001) in the rAI. We did not observe any significant between-group differences in tCho/tCr and NAA/tCr. The blood ammonia level was correlated with Glx/tCr (*r* = 0.405, *P* = 0.022) and mI/tCr (*r* = −0.398, *P* = 0.024) of the rAI. In addition, PHES was negatively correlated with Glx/tCr of the rAI (*r* = −0.379, *P* = 0.033).

**Conclusion:**

Metabolic disturbance of the rAI, which is associated with ammonia intoxication, might account for the neural substrate of cirrhosis-related cognitive dysfunction to some extent.

## Introduction

It has been demonstrated that cognitive impairment is a common neurological complication in patients with cirrhosis (1). Previous reports have suggested that cognitive impairment affects 30–45% of patients with liver cirrhosis (2, 3). The deficits of cognitive control, visuo-motor slowing, and attention decline are frequently found impairments of cognitive function in patients with cirrhosis (4). Cognitive conditions in patients with cirrhosis are linked to their employment and socioeconomic status as well as the burden of cirrhosis on caregivers (5). In fact, the presence of cognitive dysfunction has a detrimental role on patients' quality of life (6), leads to the poor work performance (5, 7), and results in the use of more healthcare resources (8). Cognitive impairment-related medical expenses also severely affect patients' activities of daily living and their adherence to therapy (9). In addition, patients with cirrhosis tend to progress from subtle cognitive dysfunction to mild hepatic encephalopathy (MHE). Additionally, MHE is prone to progress to overt hepatic encephalopathy (OHE), increasing the risk of cerebral edema, coma, and death of patients (10). The mechanisms underlying cirrhosis-related cognitive impairments, however, are not entirely understood. To identify clues for its early diagnosis and subsequent treatment, it is essential to clarify the mechanisms underlying the neurocognitive impairments (11).

The right anterior insula (rAI) is connected to the anterior cingulate as well as to frontal and anterior temporal areas (12). The rAI is responsible for cognitive function, including cognitive control processes, such as interference resolution, response selection, conflict and error monitoring, stimulus-driven decision making, and working memory (13, 14). Previous functional magnetic resonance imaging (fMRI) studies have suggested that rAI activation was associated with cognitive control capacity, including interference resolution and error processing (15, 16). Recently, other fMRI experiments have shown when rAI is activated it coactivates the autonomic system, which is also associated with response conflict monitoring and error detection (17, 18). The rAI is an important node within the salience network (SN) (19), which engages in conflict monitoring and decision making (20, 21). The central-executive network (CEN) mediates response selection and judgment in goal-directed behavior (22, 23), and the default-mode network (DMN) mediates cognitive functions, such as decision making, working memory, attentional performance, and impulsivity (24). Notably, rAI plays a critical role in switching between the CEN and DMN.

Cirrhosis has been shown to induce injury in the rAI (25). For example, neuropathological evidence has confirmed that liver cirrhosis contributes to the degeneration and death of neuronal cells in rAI (26). In addition, through *in vivo* studies using MRI, the macrostructural atrophy [reflected by cortical thinning (27)] and reduced microstructural integrity [reflected by decreased parameters derived from diffusion kurtosis imaging (28)] in rAI have been revealed in the patients with cirrhosis. Moreover, previous fMRI studies have reported that the functional connectivity of the rAI was increased in patients with cirrhosis (29, 30). Additionally, the enhanced functional connection of the rAI may be associated with a compensatory mechanism for functional damage in patients with cirrhosis who have high-level cognition (30). Furthermore, decreased glucose utilization determined by ^18^F-fluorodeoxyglucose positron emission tomography has been observed in rAI among patients with cirrhosis, which reduced performance in attention-demanding tasks, such as trail making and digit-symbol neuropsychologic tests (31). Together, cirrhosis is demonstrated to induce injury in the rAI, which may cause neurologic complications associated with cirrhosis-related cognitive dysfunction (32).

Magnetic resonance spectroscopy (MRS) has been used extensively as a non-invasive tool to evaluate brain metabolic changes in patients with cirrhosis (33, 34). For example, previous ^1^H-MRS studies have focused on anterior cingulate gyrus; frontal, parietal, and occipital lobes; and the basal ganglia (35–37). The results of these studies have almost uniformly shown an elevation in the ratio of glutamine plus glutamate compounds to creatine and decrease in choline to creatine and myo-inositol to creatine ratios (35). The ^31^P-MRS studies have focused on occipital lobes and have reported that the brain phosphocreatine was significantly reduced in cirrhosis and inorganic phosphate was increased. These results indicate a derangement of brain energy metabolism (38, 39). More important, these metabolic changes were associated with the severity of patients' cognitive dysfunction (35–39).

Although the neuropathological processes occurring in rAI may underlie the crucial mechanisms for cirrhosis-related cognitive impairment, to the best of our knowledge, the metabolic changes in rAI have not yet been explored. Thus, in this study, we applied ^1^H-MRS to observe the changes of brain metabolites in rAI in patients with cirrhosis and examined the association between these changes and patients' cognitive dysfunction.

## Materials and methods

### Subjects

This study was approved by the local research ethics committee and each participant provided written informed consent before enrollment. We selected 32 patients with cirrhosis without OHE and 31 healthy controls (HCs) to participate in this study. Patients were excluded if they (1) took psychotropic medications or had a history of drug abuse; (2) had abused alcohol in the 6 months before the study; (3) had an uncontrolled endocrine or metabolic disorder, such as thyroid dysfunction; and (4) currently had OHE or another neuropsychiatric condition. We employed the West Haven criteria (40) to determine whether or not the patients had overt symptoms of HE. We used the psychometric hepatic encephalopathy score (PHES) to determine neurocognitive function (41). The assessment included a digit symbol test, number connection tests A and B, line tracing test, and serial dotting test.

### MRI data acquisition

**Figure 1:**
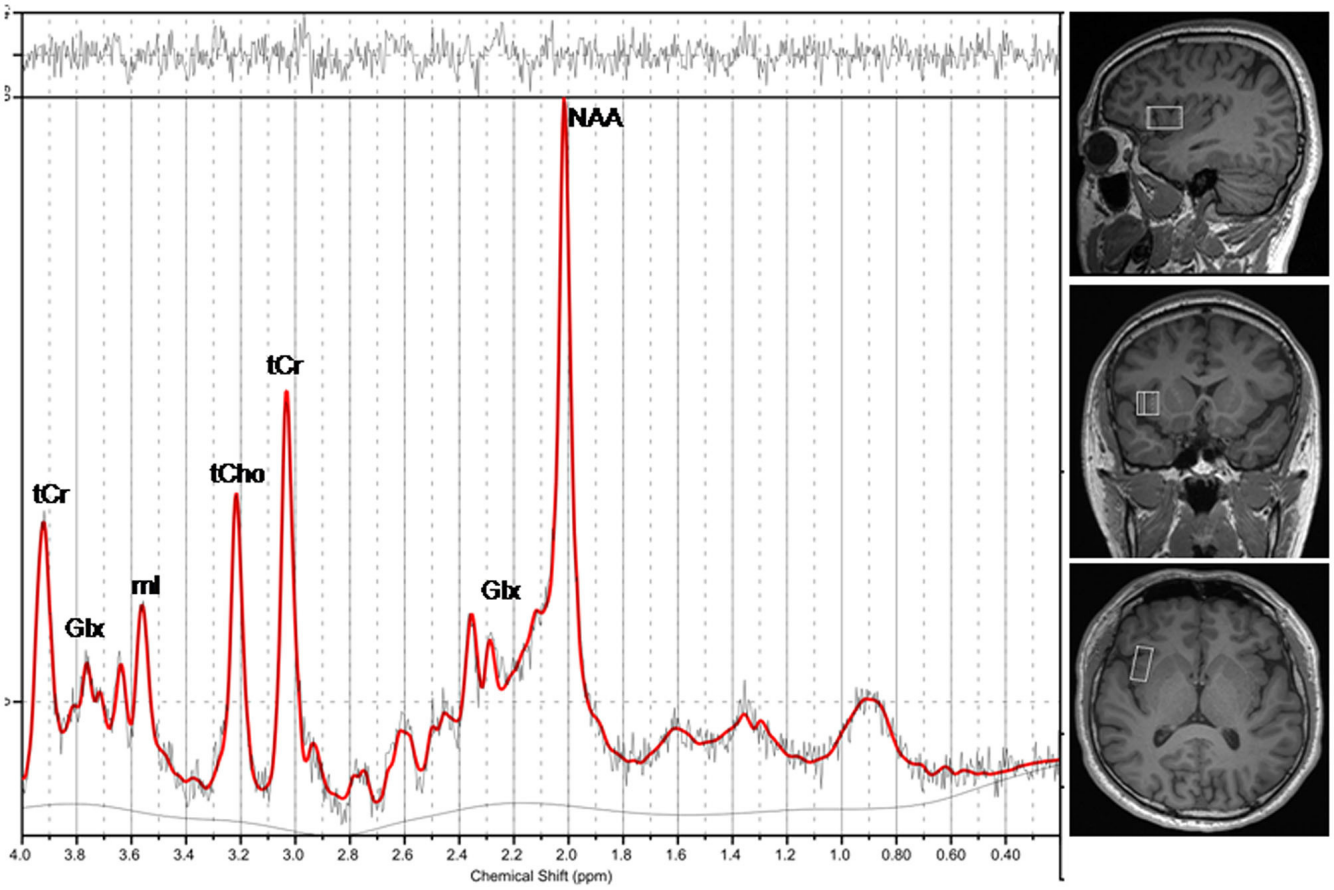
A representative magnetic resonance spectroscopy of the rAI in a healthy individual. NAA, N-acetylaspartate; Glx, glutamate plus glutamine; mI, myo-inositol; tCho, total choline; tCr, total creatine.

We performed MRI using a 3T MRI scanner (Magnetom Prisma, Siemens Healthcare, Erlangen, Germany) with 64-channel head coil. We first acquired three-dimensional high-resolution T1-weighted images. The spectroscopic data acquisition included image-guided localization of the volume of interest (VOI) according to the following parameters: repetition time (TR) = 1,610 ms, inversion time = 900 ms, echo time (TE) = 2.25 ms, field of view = 224 mm × 224 mm, matrix = 224 × 224, flip angle = 8°, slice thickness = 1.0 mm, and 176 sagittal slices. To obtain spectroscopic data, we used single-voxel point-resolved ^1^H-MRS sequence according to the following parameters: TR = 2,000 ms, TE = 30 ms, number of averages = 72, flip angle = 90°, vector size = 1,024, and bandwidth = 1,200 Hz. A VOI with size of 2.5 cm × 1.5 cm × 1.0 cm was selected on the rAI (Figure 1). Before imaging, we used automated procedures to perform shimming and tuning and suppressed water signals according to a chemical shift selective suppression algorithm.

### MRS data processing

The MRS raw data files with RDA format were exported from the scanner, within that coil combination and spectral averaging were performed. Subsequently, the MR spectra were automatically processed using LCModel software (6.3-1L, S. Provencher Inc., Oakville, ON, Canada). The zero-order and first-order phase correction were performed for the MR spectra. Fitting was carried out between 0.2 and 4.0 ppm. To ensure the reliability of the results, visual assessment was performed on the spectra fitting result (42). Furthermore, quantitative quality assessment indices, including a signal-to-noise ratio (SNR) > 3.0 (42), a full width at half-maximum (FWHM) for NAA <0.1 ppm (42), and Cramer-Rao lower bounds (CRLB) below 20%, were considered in the analysis. MRS data reporting followed standardized guidelines (43) (Supplementary Table 1). Assignment of the resonances of interest included N-Acetylaspartate (NAA) at 2.01 ppm; glutamate and glutamine (Glx) at about 3.75 ppm and between 2.04 and 2.46 ppm; total creatine (tCr) at 3.03 ppm and 3.91 ppm combined creatine (Cr) and phosphocreatine (PCr); total choline (tCho) at about 3.20 ppm, which was contributed to free choline (Cho), glycerophoshorylcholine (GPC), and phosphorylcholine (PCh); and myo-inositol (mI) at about 3.55 ppm (Figure 1). We calculated relative concentrations of brain metabolites as ratios to tCr for its relatively stable concentration in the brain (44, 45), including NAA/tCr, Glx/tCr, tCho/tCr, and mI/tCr.

In addition, the proportion of different tissues within the VOI was determined, after the T1 weighted images had been segmented into white matter (WM), gray matter (GM), and cerebrospinal fluid (CSF) using Statistical Parametric Mapping 8 software (SPM8, http://www.fil.ion.ucl.ac.uk/spm).

### Statistical analysis

To compare intergroup demographic variables, such as age and years of education; neurocognitive performance, such as PHES; and metabolite concentration, including NAA/tCr, Glx/tCr, tCho/tCr, and mI/tCr, we adopted non-parametric Mann–Whitney *U*-test. Also, we compared the proportions of GM, WM, and CSF within the VOI between the groups using the Mann-Whitney *U*-Test. We applied chi-squared test for intergroup comparison of categorical variables (i.e., gender). Furthermore, we performed the Spearman correlation analyses between clinical parameters (including blood ammonia level, Child-Pugh score, and PHES) and metabolite concentration among the patients with cirrhosis. We set statistical significance at *P* < 0.05.

## Results

Table 1 lists the clinical and demographic characteristics of study participants. We did not observe any statistical differences between the patients with cirrhosis and HCs in terms of sex, age, and years of education. The etiology of cirrhosis included HBV (65.6%), HBV and alcoholism (15.6%), alcoholism (12.5%), and cryptogenic cause (6.3%). The mean Child-Pugh score of cirrhosis patients was 6.6 ± 1.6 points; and there were 18 Child-Pugh A (5 – 6 points), 13 Child-Pugh B (7 – 9 points), and 1 Child-Pugh C (10 – 15 points) patients (46). Child-Pugh A represents well-compensated cirrhosis, Child-Pugh B represents cirrhosis with significant functional compromise, and Child-Pugh C represents decompensated cirrhosis (46). In patients with cirrhosis, the mean blood ammonia level was 32.7 ± 19.8 μmol/L (normal reference range for healthy population is 9 – 33 μmol/L). Additionally, the patients with cirrhosis performed worse on the PHES assessment (0.4 ± 1.5 vs. −1.6 ± 2.4, *P* < 0.001) compared with HCs.

**Table 1:** Study participant clinical and demographic characteristics.

	**Healthy controls (*n* = 31)**	**Patients with cirrhosis (*n* = 32)**	***P*-value**
Age (years)	52.8 ± 7.1	49.1 ± 10.6	0.148
Sex (male/female)	26/5	30/2	0.257
Years of education	8.3 ± 3.3	7.3 ± 3.5	0.250
**Etiology of cirrhosis**			
Hepatitis B virus (HBV)		21 (65.6%)	
Alcoholism	-	4 (12.5%)	-
HBV and alcoholism		5 (15.6%)	
Cryptogenic cause		2 (6.3%)	
Child-Pugh score	-	6.6 ± 1.6	-
Blood ammonia level (μmol/L)	-	32.7 ± 19.8	-
Psychometric hepatic encephalopathy score	0.4 ± 1.5	−1.6 ± 2.4	<0.001

The quantitative quality assessment indicators for the final selected MRS data are shown in the Supplementary Table 2. The GM ratio (0.74 ± 0.09 vs. 0.70 ± 0.10, *P* = 0.112), WM ratio (0.06 ± 0.10 vs. 0.06 ± 0.11, *P* = 0.114), and CSF ratio (0.20 ± 0.06 vs. 0.24 ± 0.09, *P* = 0.141) in the VOI showed no significant differences between groups, indicating consistent voxel placement.

**Figure 2:**
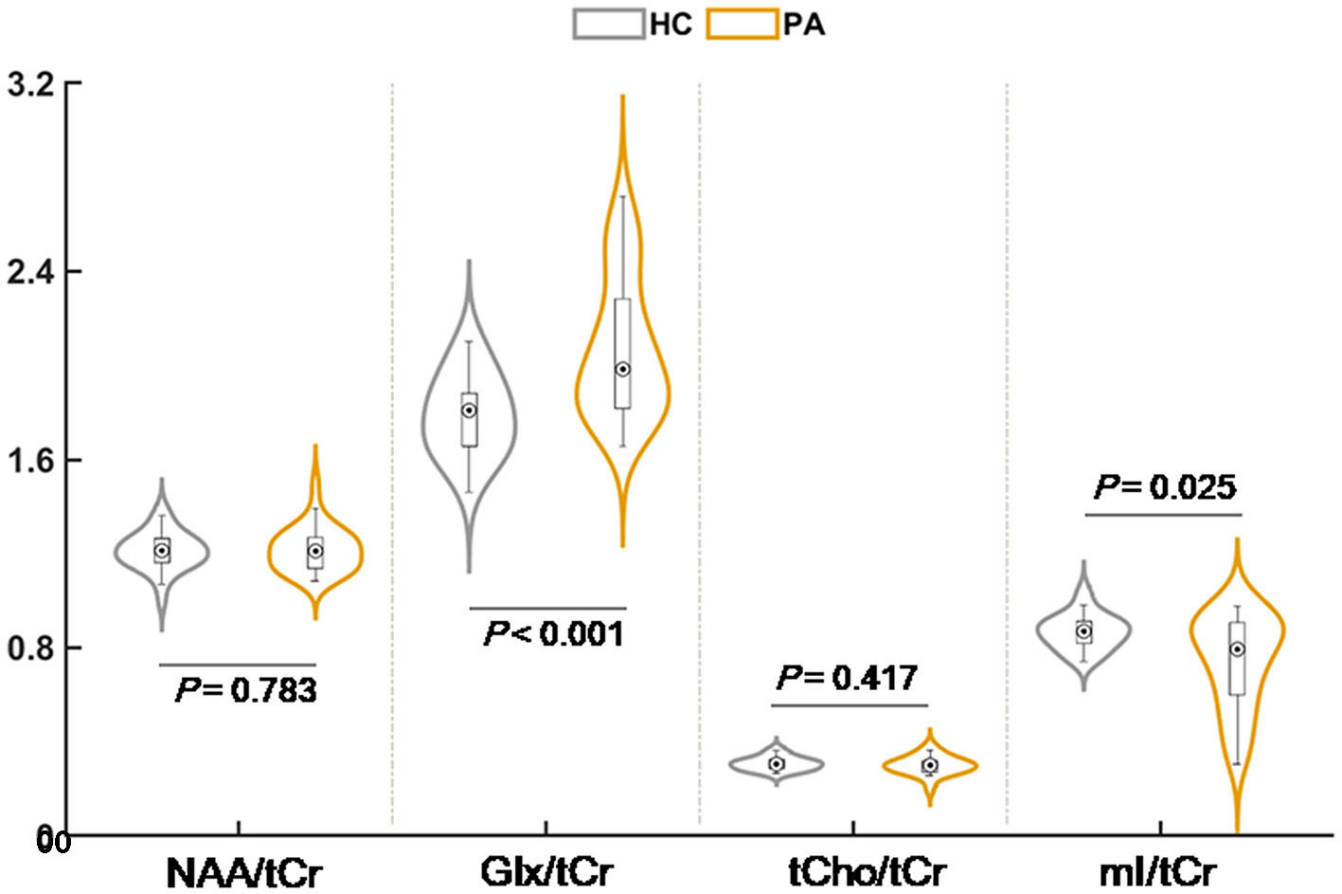
The result of intergroup comparison for relative metabolite concentration. NAA, N-acetylaspartate; Glx, glutamate plus glutamine; mI, myo-inositol; tCho, total choline; tCr, total creatine.

Figure 2 and Table 2 displays the MRS results. Compared with HC, the patients with cirrhosis exhibited significantly increased Glx/tCr (1.79 ± 0.17 vs. 2.07 ± 0.29, *P* < 0.001) and decreased mI/tCr (0.87 ± 0.07 vs. 0.74 ± 0.19, *P* = 0.025) in the rAI. We did not observe any significant between-group differences in NAA/tCr (1.21 ± 0.08 vs. 1.22 ± 0.09, *P* = 0.783) and tCho/tCr (0.31 ± 0.03 vs. 0.30 ± 0.04, *P* = 0.417) in the rAI.

**Table 2:** Relative metabolite concentration difference between groups.

	**Healthy controls (*n* = 31)**	**Patients with cirrhosis (*n* = 32)**	***P*-value**
Glx/tCr	1.79 ± 0.17	2.07 ± 0.29	<0.001
mI/tCr	0.87 ± 0.07	0.74 ± 0.19	0.025
NAA/tCr	1.21 ± 0.08	1.22 ± 0.09	0.783
tCho/tCr	0.31 ± 0.03	0.30 ± 0.04	0.417

**Figure 3:**
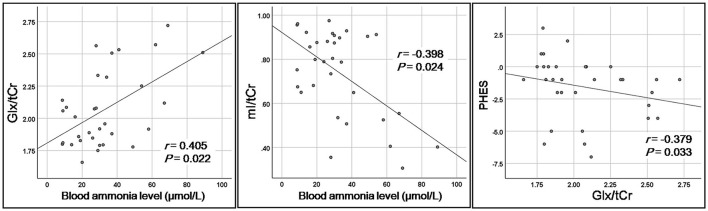
Correlations between clinical parameters and metabolic measurements.

The association between clinical parameters and metabolite concentration is shown in Figure 3 and Table 3. The correlation was positive for the Glx/tCr of the rAI and the blood ammonia level (*r* = 0.405, *P* = 0.022), and the correlation was negative between the mI/tCr of the rAI and blood ammonia level (*r* = −0.398, *P* = 0.024). In addition, the Glx/tCr of the rAI was negatively correlated with PHES (*r* = −0.379, *P* = 0.033).

**Table 3:** Correlations between clinical parameters and metabolic measurements.

	**Correlations with blood ammonia level**	**Correlations with PHES**
	***r, P***	***r, P***
Glx/tCr	0.405, 0.022	−0.379, 0.033
mI/tCr	−0.398, 0.024	0.192, 0.292
NAA/tCr	0.057, 0.756	0.054, 0.771
tCho/tCr	−0.050, 0.784	0.208, 0.254

## Discussion

In this study, we explored cirrhosis-related neurochemicals alteration in the rAI using ^1^H-MRS and determined its association with clinical parameters. The primary findings were as follows: First, patients with cirrhosis showed poor performance on the PHES, indicating that cognitive function was impaired. Second, we observed increased Glx/tCr and decreased mI/tCr in patients with cirrhosis, which reflected a metabolic disturbance in the rAI. Third, we observed an association between the Glx/tCr and mI/tCr of the rAI and blood ammonia level, which suggested that MRS alteration in patients with cirrhosis might be related to ammonia intoxication. Fourth, patients with cirrhosis showed a negative correlation between the Glx/tCr of the rAI and PHES, which indicated that metabolic disturbance in the rAI might be responsible for cognitive impairment.

In the current study, we found increased Glx/tCr and decreased mI/tCr in the rAI in patients with cirrhosis, indicating the elevation of glutamine and glutamate and reduction of mI in the brain tissue. Consistently, previous MRS studies on cirrhosis have revealed similar metabolic alterations in other brain regions (such as the basal ganglia, parietal white matter, occipital gray matter, and anterior cingulate gyrus) (47–49). In addition, as demonstrated in a series of studies, the rAI is indeed a vulnerable node that is affected by hepatic cirrhosis. In fact, altered spontaneous brain activities in the rAI and decreased functional connectivity between the rAI and right supramarginal gyrus also have been observed (30, 50), which are in agreement with our findings. Given that the rAI is a pivotal hub within the SN and coordinates and facilitates the activities of high-level cognition networks (such as the DMN and CEN) (51), we inferred that the disturbed metabolism in the rAI may be associated with impairment of multiple cognitive domains (such as cognitive control, attentional process, and decision making) (19, 52). This speculation was supported by the observed correlation between worse cognitive performance and increased Glx/tCr in the rAI in our study.

Hyperammonemia is a common event associated with cirrhosis (53). Previous studies have suggested that cirrhosis-related MRS alterations (i.e., increased Glx/tCr and decreased mI/tCr) are attributed to ammonia intoxication (54, 55). In astrocytes, the glutamine synthetase is a major glutamate-metabolizing enzyme that is involved in ammonia detoxification and metabolizes glutamate to glutamine (56, 57). In case of hyperammonemia, more ammonia crosses the blood-brain barrier and is converted to glutamine through glutamine synthetase in astrocytes, which lead to glutamine accumulation in the brain (57). Glutamine (a powerful substance that is osmotically active) elicits astrocyte swelling by drawing extracellular water into the astrocytes (58). To counterbalance the osmotic effect of cerebral glutamine accumulation, mI (another astrocytic osmolyte) was rapidly decreased to protect the brain from massive edema (59). In this context, it is reasonable that we can detect increased Glx/tCr and decreased mI/tCr ratios in the rAI. The results of our correlation analysis, which showed an association between blood ammonia level and metabolism disturbance (i.e., increased Glx/tCr and decreased mI/tCr ratios in the rAI), provides supporting evidence for our inference.

We also found that an imbalance of neurotransmitters in the brain was associated with disturbed metabolism patterns of the rAI in patients with cirrhosis (36). As the primary excitatory neurotransmitter in the central nervous system, glutamate is responsible for cognition processes, such as memory formation and retrieval, information encoding, consciousness maintenance, and spatial recognition. The regulation of the glutamate-glutamine cycle affects glutamate homeostasis (60). During the occurrence of hyperammonemia in patients with cirrhosis, the glutamate-glutamine cycle was deregulated because of the blocking of the glutamate excitatory amino-acid transporters 1 and 2, leading to the disturbance of glutamate homeostasis (61). Additionally, mI plays a crucial role in phospholipid metabolism as a second messenger in the phosphatidylinositol cycle and is involved in calcium-mediated glutamatergic signaling (62). mI depletion of the rAI in patients with cirrhosis may contribute to glutamatergic signaling disturbance. Together, the increase in Glx/tCr and decrease in mI/tCr may be indicators for glutamatergic neurotransmitter dysfunction, which may be responsible for cognitive impairment in patients with cirrhosis.

Several limitations regarding this study deserve attention. First, we analyzed the concentration of metabolites relative to tCr in this study, which is a commonly used method in MRS (63, 64). However, it has been observed that tCr levels vary in different neurological diseases (65, 66), and creatine levels exhibited an upward trend with aging (67), thus cautions are required in interpreting results. In order to minimize the impact of aging on the results, the age of the two groups in this study was matched. Obtaining metabolite concentration using unsuppressed water peak as a reference is an alternative approach. Cerebral water content is relatively uniformly distributed, and pathological changes in water content vary over a relatively small range (68), which can lead to more accurate quantification. This could be a promising direction in our future study. Second, this study was cross-sectional. A longitudinal study should be performed to observe the metabolic alteration of the rAI following treatment, to verify the causal relationship between blood ammonia alteration and MRS changes. Third, we did not conduct a more comprehensive analysis to assess the effect of potential confounding factors (e.g., etiology, gender, and body mass index) on the results. Last, similarly to other studies (34, 69, 70), we did not measure blood ammonia for healthy controls, which limits the comparison between groups.

## Conclusion

In conclusion, the disturbed metabolism in the rAI might be one of the characteristics in cirrhosis. Metabolism disturbance in the rAI, which was correlated with ammonia intoxication, might account for the neural substrate of cirrhosis-related cognitive dysfunction to some extent. Further multicenter research with large sample sizes is warranted to validate our conclusion.

## Data availability statement

The original contributions presented in the study are included in the article/Supplementary material, further inquiries can be directed to the corresponding authors.

## Ethics statement

The studies involving humans were approved by Ethics Committee of Fujian Medical University Union Hospital. The studies were conducted in accordance with the local legislation and institutional requirements. The participants provided their written informed consent to participate in this study.

## Author contributions

N-XH: Data curation, Writing – original draft. H-WH: Data curation, Writing – original draft. Q-YD: Writing – original draft, Data curation. Y-LW: Methodology, Writing – original draft. DL: Writing – review & editing, Data curation, Investigation. J-QL: Methodology, Writing – review & editing. H-JC: Conceptualization, Data curation, Formal analysis, Funding acquisition, Investigation, Methodology, Supervision, Validation, Visualization, Writing – review & editing.
