# Supplementary material for: Metabolic alterations in the right anterior insula among patients with cirrhosis without overt hepatic encephalopathy: a magnetic resonance spectroscopy study
Source: Front Neurol. 2024 Jan 11;14:1291478. doi: 10.3389/fneur.2023.1291478 (PMC10811796; doi:10.3389/fneur.2023.1291478)
Supplement: Supplementary file 1 [file Table_1.pdf]

Supplementary Table 1: Minimum Reporting Standards for in vivo MR Spectroscopy

**MRSinMRS checklist**	
1. Hardware	
a. Field strength [T]	3T MRI scanner
b. Manufacturer	Siemens Healthcare
c. Model (software version if available)	Magnetom Prisma
d. RF coils: nuclei (transmit/ receive), number of channels, type, body part	^{1}H, 64 channel head coil
e. Additional hardware	N/A
2. Acquisition	
a. Pulse sequence	Point-Resolved Spectroscopy (PRESS)
b. Volume of Interest (VOI) locations	Right anterior insula (rAI)
c. Nominal VOI size [cm^3, mm^3]	$2.5 \times 1.5 \times 1.0$ cm^3
d. Repetition Time (TR), Echo Time (TE) [ms, s]	TR = 2000 ms, TE = 30 ms
e. Total number of Excitations or acquisitions per spectrum<br>In time series for kinetic studies<br>i. Number of Averaged spectra (NA) per time-point<br>ii. Averaging method (e.g. block-wise or moving average)<br>iii. Total number of spectra (acquired / in time-series)	72 averages per spectrum with constant acquisition parameters
f. Additional sequence parameters (spectral width in Hz, number of spectral points, frequency offsets);<br>If STEAM: Mixing Time TM;<br>If MRSI: 2D or 3D, FOV in all directions, matrix size, acceleration factors	Bandwidth 1200 Hz, 1024 spectral points
g. Water Suppression Method	Chemical Shift Selective (CHESS) method
h. Shimming Method, reference peak, and thresholds for "acceptance of shim" chosen	Automated B0 field mapping
i. Triggering or motion correction method (respiratory, peripheral, cardiac triggering, incl. device used and delays)	N/A
3. Data analysis methods and outputs	
a. Analysis software	LCModel software (vers 6.3-1L)
b. Processing steps deviating from quoted reference or product	N/A
c. Output measure (e.g. absolute concentration, institutional units, ratio)	Ratio to total creatine
d. Quantification references and assumptions, fitting model assumptions	Basis: press _te30_3t_gsh_v3. basis (provided by LCModel)
4. Data Quality	
a. Reported variables (SNR, Linewidth (with reference peaks))	SNR[a], FWHM[b] (see Supplementary Table 2)
b. Data exclusion criteria	Spectra with poor visual fit; CRLB[c] > 20%; SNR < 3[d]; FWHM > 0.1ppm[d]
c. Quality measures of postprocessing Model fitting (e.g. CRLB, goodness of fit, SD of residual)	CRLB (see Supplementary Table 2)
d. Sample Spectrum	See Figure 1

Supplementary Table 2: Spectral and fitting quality metrics for each investigated metabolite in study group.

Quality index	Mean ± Std (minimum - maximum)	
	Healthy controls	Patients with cirrhosis
$CRLB_{Ins}$ [%]	4.39 ± 0.62 (3 - 6)	5.88 ± 2.42 (4 - 16)
$CRLB_{tCho}$ [%]	3.06 ± 0.25 (3 - 4)	3.16 ± 0.37 (3 - 4)
$CRLB_{tCr}$ [%]	2.58 ± 0.50 (2 - 3)	2.81 ± 0.40 (2 - 3)
$CRLB_{NAA}$ [%]	3.10 ± 0.30 (3 - 4)	3.31 ± 0.47 (3 - 4)
$CRLB_{Glx}$ [%]	6.32 ± 0.94 (4 - 9)	6.06 ± 1.08 (4 - 9)
SNR	19.32 ± 2.39 (15 - 25)	18.38 ± 4.29 (12 - 37)
$FWHM_{NAA}$ [ppm]	0.037 ± 0.005 (0.029 - 0.048)	0.038 ± 0.006 (0.029 - 0.052)

a. SNR: the ratio of the maximum in the spectrum-minus Baseline over the Analysis Window (0.2-4) to twice the rms Residuals.
b. FWHM: full width at half-maximum of the NAA.
c. CRLB: Cramer-Rao lower bounds.
d. Threshold recommended by consensus [1].

[1] M. Wilson, O. Andronesi, P.B. Barker, R. Bartha, A. Bizzi, P.J. Bolan, K.M. Brindle, I.Y. Choi, C. Cudalbu, U. Dydak, U.E. Emir, R.G. Gonzalez, S. Gruber, R. Gruetter, R.K. Gupta, A. Heerschap, A. Henning, H.P. Hetherington, P.S. Huppi, R.E. Hurd, K. Kantarci, R.A. Kauppinen, D.W.J. Klomp, R. Kreis, M.J. Kruiskamp, M.O. Leach, A.P. Lin, P.R. Luijten, M. Marjanska, A.A. Maudsley, D.J. Meyerhoff, C.E. Mountford, P.G. Mullins, J.B. Murdoch, S.J. Nelson, R. Noeske, G. Oz, J.W. Pan, A.C. Peet, H. Poptani, S. Posse, E.M. Ratai, N. Salibi, T.W.J. Scheenen, I.C.P. Smith, B.J. Soher, I. Tkac, D.B. Vigneron, F.A. Howe, Methodological consensus on clinical proton MRS of the brain: Review and recommendations, Magn Reson Med 82(2) (2019) 527-550.
